# Investigation of the hydrophobic and acoustic properties of bio windmill palm materials

**DOI:** 10.1038/s41598-018-31691-0

**Published:** 2018-09-07

**Authors:** Changjie Chen, Zhong Wang, You Zhang, Ming Bi, Kaiwei Nie, Guohe Wang

**Affiliations:** 1 College of Textile and Clothing Engineering, Soochow University, Suzhou, 215006 Jiangsu China; 2 Nantong Textile & Silk Industrial Technology Research Institute, Nantong, 226108 Jiangsu China

## Abstract

Windmill palm fibers are an abundant lignin-cellulose fiber resource. Single palm fibers can be prepared using an alkali treatment method. However, these fibers have hydrophilic surfaces, and following drying the fibers exhibit serious aggregation. This limits their application as acoustic materials. In this work, both alkali and acetylation treatments were used to modify the characteristics of windmill palm fibers. These treatments caused the surface of the fibers to become hydrophobic and increased the specific area and free vacuum space of the fibers, thus lowering energy loss. Scanning electron microscope observations combined with Fourier-transform infrared spectroscopy showed that the acetylation treatment resulted in the substitution of hydroxyl groups with acetyl groups, and the formation of nanoscale pores (10~50 nm). The results of the moisture-absorption and contact-angle tests showed that the moisture regain value decreased to 3.86%, and the contact angle increased to above 140° after acetylation treatment. The average sound absorption coefficients of the alkalized and acetylated nonwoven fabrics were 0.31 and 0.36, respectively. The masses of the acetylated samples were 50% those of the windmill palm sheath samples.

## Introduction

With growing global environmental awareness, there has been increased focus on the development of environmentally friendly bio-resources^1–4^. Windmill palm is one of the most cultivated palms in China. Single windmill palm fibers can be extracted from this raw cellulose-material resource. Basic information on the windmill-palm fiber, as well as the single fibers, is shown in Table 1^5^. However, to date, this abundant, inexpensive, and biocompatible cellulose resource has not been utilized commercially^6,7^. Short, single, windmill-palm fibers, with an average length of about 600 μm, are not suitable for clothing purposes, and are therefore generally discarded as waste^5^. However, a lumen exists in the center of these windmill-palm fibers; this hollow structure can increase the friction that exists between sound waves and the fibers. Therefore, these windmill-palm fibers could potentially be applied to prepare nonwoven fabrics with good acoustic properties^8^. While, high levels of noise pollution can cause various types of public health hazards, such as deafness, and even heart ailments^9^.

**Table 1:** The chemical compositions and physical structures of the windmill palm fiber and single fiber.

Samples	Chemical composition/%				Physical structure/μm		
	Ash	Lignin	α-Cellulose	Hemi-Cellulose	Length	Diameter	Hollowness /%
Windmill palm fiber	1.23	23.52	52.26	19.23	3.71 × 10^5^	400	—
Single fiber	0.54	7.24	78.18	11.32	637.96	10.01	47.21

The acetylation treatment process is attractive and simple, and is widely applied to modify the hydroxyl (OH) groups on the cellulose surface^10,11^. The key to this method is to replace the OH groups of the fiber with acetyl (CH_3_CO) groups. Many researchers use this treatment to improve the fiber–matrix adhesion of cellulose fiber/polymer composites^8,12^. However, there have been no reports on the use of waste windmill-palm fibers as raw materials for the development of hydrophobic materials, especially with excellent sound-absorption properties.

In this study, alkalized and acetylated single windmill-palm fibers were prepared and their microstructure, hydrophobic properties, and acoustic properties were investigated. Such acetylation treatments are considered to be a novel method for the preparation of lightweight, nonwoven fabrics with good acoustic performances.

## Results

### Acetylation treatment and hydrophobic properties

Prior to the orthogonal design, single-factor tests were conducted. At the start of the treatment, the contact angle increases with the acetyl chloride concentration, pyridine concentration, temperature, and time, respectively. Subsequently, the contact angle slightly decreases as the reagent concentration further increases. Consequently, an orthogonal design involving four-factor and three-level experiment was used to optimize the parameters used for the modification of the windmill palm fiber, in which the fiber would develop a hydrophobic surface. The details are shown in Table 2.

**Table 2:** Factors and levels of the orthogonal design.

Level	Factor			
	A acetylchloride/vt.%	B pyridine/vt.%	C Temperature/°C	D Time/h
1	7.5	2.5	30	4
2	12.5	5.0	40	6
3	17.5	7.5	50	8

The effects of the four factors on the contact angle of the windmill-palm fiber are shown in Table 3. As shown, factor A (concentration of acetyl chloride), with the highest range (R), has the greatest influence on the contact angle. In addition, the temperature, with the smallest R, only has a slight influence on the hydrophobic properties of the windmill palm fiber. As shown in the Table 3, the parameters with the highest K (the sum of contact angle) values can be considered optimal. In general, the optimal parameters for modification of the natural windmill palm fibers can be considered to be 12.5 vt.% acetyl chloride, 7.5 vt.% pyridine, temperature of 50 °C, and treatment time of 8 h.

**Table 3:** The characteristics of the fibers treated under various conditions.

Samples	Factor Result				
	A	B	C	D	Contact angle/°
1	1	1	1	1	126.48
2	1	2	2	2	133.28
3	1	3	3	3	136.08
4	2	1	2	3	136.95
5	2	2	3	1	141.25
6	2	3	1	2	142.35
7	3	1	3	2	133.78
8	3	2	1	3	140.58
9	3	3	2	1	139.70
K_1_	395.83	397.20	409.40	407.43	
K_2_	420.55	415.10	409.93	409.40	
K_3_	414.05	418.13	411.10	413.60	
R	24.73	20.93	1.70	6.17	

**Figure 1:**
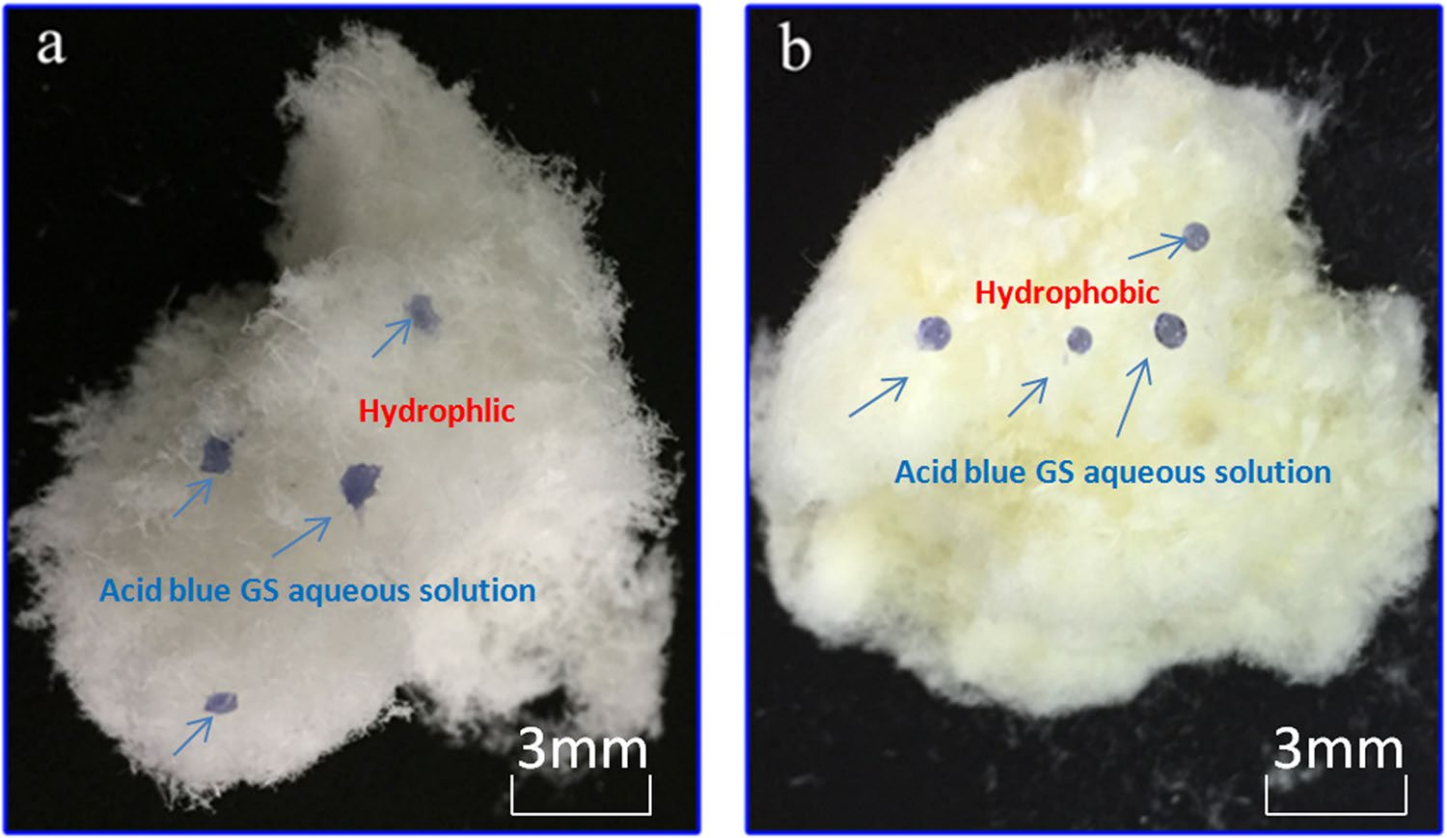
Images of **(a**) alkalized single windmill palm fiber, and (**b**) acetylated single fiber.

Following the treatment under the optimal parameters, the static water-contact angles of the windmill-palm fiber increased from 20 ± 6° to 143 ± 4°. In addition, Fig. 1 shows that the nature of the surface changed from hydrophilic to hydrophobic. The acetylation treatment replaces the OH groups on the cell walls of the windmill palm fibers with CH_3_CO groups, which decreases the number of OH groups present on the fiber surface. This causes the hydrophilic windmill palm fibers to transform into hydrophobic fibers.

### Morphological investigation of the alkalized and acetylated windmill palm fibers

**Figure 2:**
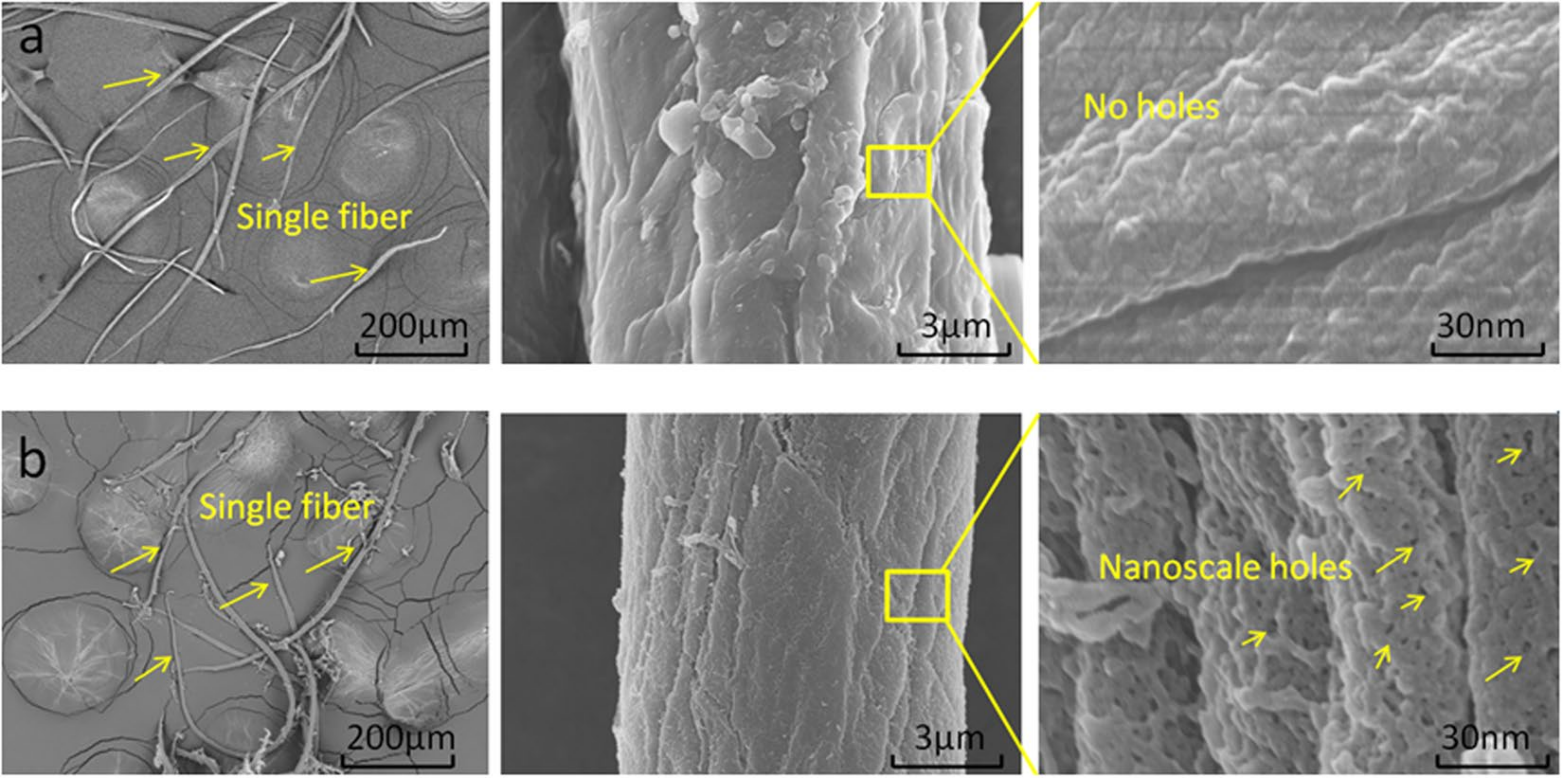
Surface morphologies of (**a**) alkalized single windmill palm fiber, and (**b**) acetylated fiber.

The lignocellulose windmill palm fiber is a natural composite that is primarily composed of cellulose, hemicellulose, and lignin. The cellulose, which has a high degree of crystallinity, has an important function of reinforcement^13^. Owing to hydrolysis, hemicellulose is soluble in alkali, and compared with cellulose, is expected to exhibit poor stability in dilute alkali solutions. This is because the hemicellulose possesses a branched structure while the cellulose has a linear structure^14,15^. Lignin can be solubilized via alkali treatments. This improves the solubility of the lignin in the solution^16^. The alkalization of the windmill palm fibers resulted in the hydrolysis of the composite fiber bundles, forming single fibers. This was due to the solubilization of the hemicellulose and the partial degradation of the lignin. The single fibers consist of hydrophilic cellulose fibers that each possess a large lumen in the center of their cross section^5^. The change in the morphology of the alkalized, single windmill palm fiber following the acetylation treatment is shown in Fig. 2. Irregularly shaped, aggregated fibrils can be observed in Fig. 2(a). The dense surface wall of the fiber can be clearly observed even when it is by magnified ×90 K. Meanwhile, Fig. 2(b) shows that the aggregation has disappeared following the chemical treatment, and large numbers of nanoscale pores have formed. The mean pore size ranges from 10 to 50 nm.

### Chemical composition

**Figure 3:**
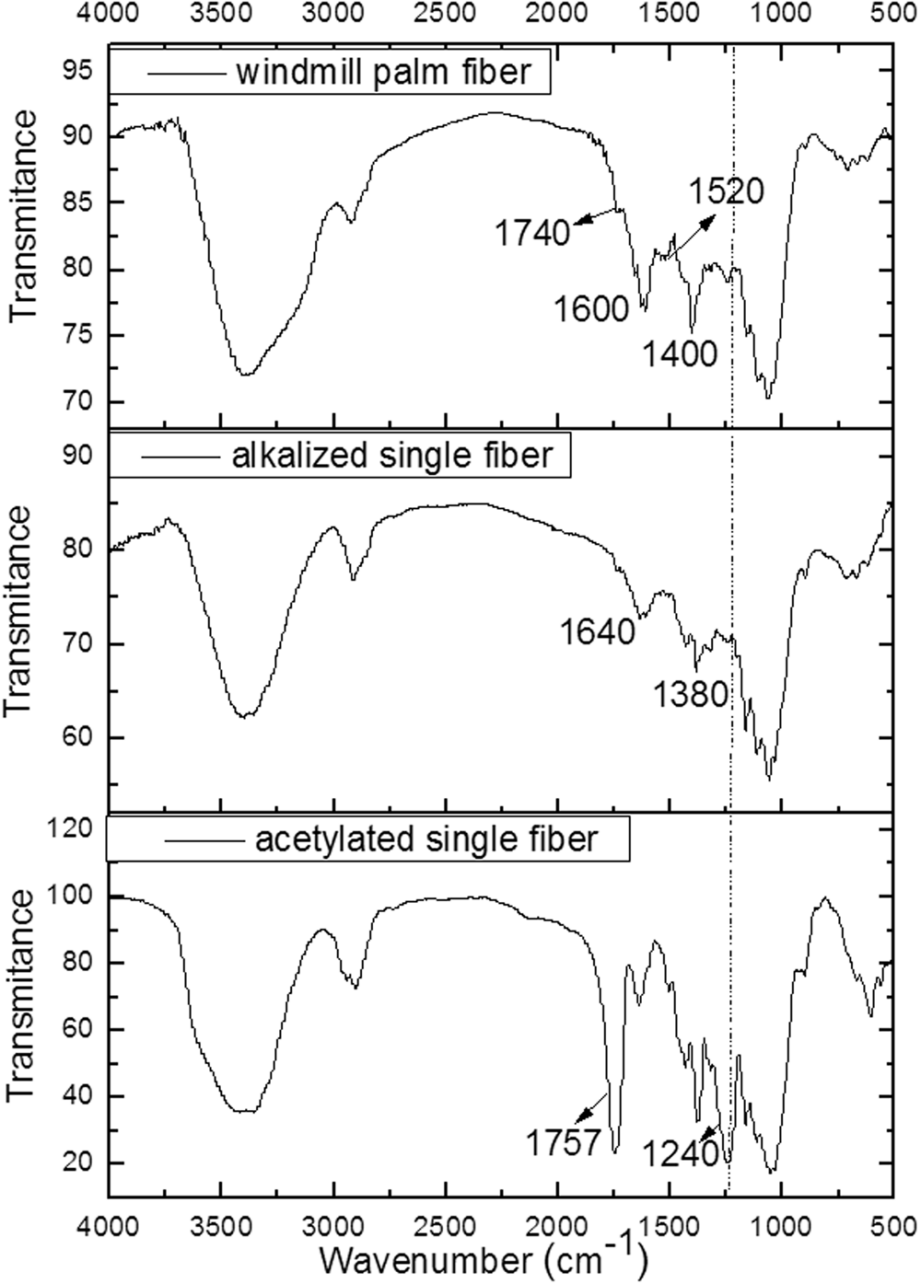
FTIR spectra of windmill palm materials.

The Fourier-transform infrared (FTIR) spectra of the windmill palm fiber, alkalized single fiber, and acetylated single fiber are shown in Fig. 3. All the windmill-palm fibers exhibited broad absorption peaks at around 3400 cm^−1^ and 2900 cm^−1^, which were attributed to OH stretching and CH stretching, respectively^17^. The lignin presented characteristic peaks over the range of 1500~1600 cm^−1^, which were attributed to the aromatic C=C stretching of the aromatic ring of the lignin^18^. In addition, the windmill palm fiber exhibited a peak at 1740 cm^−1^, which corresponded to the C=O stretching of the CH_3_CO groups of the hemicelluloses^19^. While, in the cases of the alkalized and acetylated fibers, these peaks were absent. This indicates that lignin and hemicellulose were not present in these fibers.

The peaks at around 1400 cm^−1^ can be attributed to the C-CH_3_ band^20^. The absorption peaks at 1640 cm^−1^ can be attributed to the OH stretching of the water absorbed by the cellulose^21^. In the case of the acetylated fiber, the peaks at 1757 cm^−1^ could be attributed to the C=O stretching of the carbonyl groups of the ester bonds^22^. The vibration peaks at about 1240 cm^−1^ can be attributed to the C–O stretching of the CH_3_CO groups^22^. These two peaks confirmed the acetylation of the windmill palm fiber^22^.

### Absorption properties

**Figure 4:**
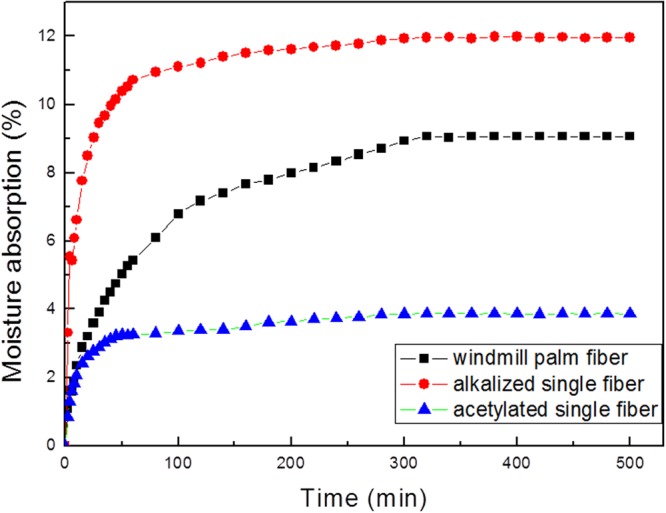
The moisture absorption curves obtained for the raw and treated windmill palm fibers.

Figure 4 presents the moisture-absorption curves obtained for the windmill palm fiber, and the alkalized and acetylated single fibers. In the cases of the three samples, the initial rate of moisture absorption is extremely rapid. The curves decrease from 30 to 300 min for the windmill palm fiber. And this decrease for alkalized fiber and acetylated fiber were from 16 to 80 min, and 10 to 50 min respectively. The raw, alkalized, and acetylated fibers all achieve moisture sorption equilibrium following a period of 5 h. The alkali treatment resulted in the removal of the hydrophobic wax and silica from around the fiber surface. The OH groups of the cellulose react with the water molecules, which contributes to the increased water absorption^23,24^. The swelling of the alkalized single fiber also contributes to the increase in the moisture absorption^25–27^. Thus, in this study, the moisture regain value of the alkalized single fiber is the highest, namely 11.96%.

The windmill palm fiber demonstrates a moisture regain value of 9.07%; this is slightly lower than that of the alkalized single fiber, but greater than those of cotton (7.48%) and flax (8.51%)^7^. As shown in Table 1 and Fig. 2, the palm fiber prepared directly from the sheath mesh exhibits low crystallinity, a rough morphology, an abundance of hollow fiber cells, and a relatively high hemicellulose content. These factors contribute to the excellent moisture absorption properties of this fiber. In the case of the acetylated fiber, the moisture regain value is extremely low, namely 3.86%.

### Acoustic properties of the windmill-palm fibers

Alkalized nonwoven windmill palm fabric, acetylated nonwoven fabric, and raw palm sheath samples, each with a thickness of about 8 mm and diameter of 10 mm, were prepared. The weights of the three material samples were 5 g, 5 g, and 10 g, respectively.

**Figure 5:**
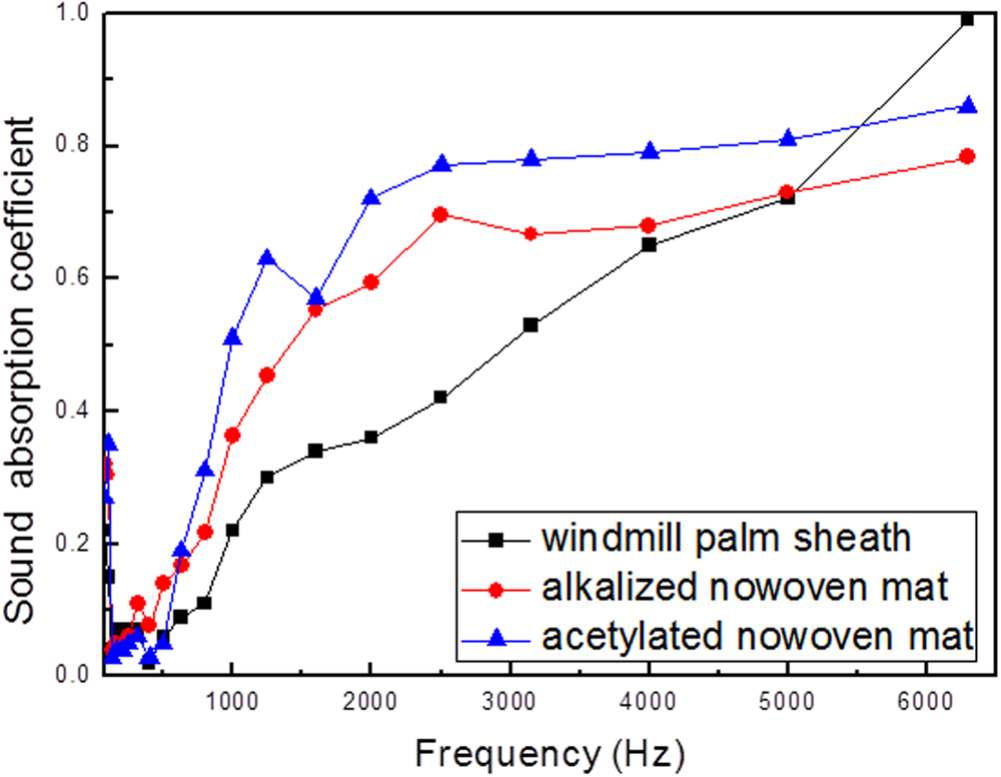
Sound absorption coefficients of the various nonwoven windmill palm fabrics.

Figure 5 presents the absorption performance of the alkalized windmill palm fiber, acetylated fiber, and palm sheath materials. As shown, amongst the samples tested, the acetylated nonwoven fabric exhibits relatively high levels of absorption. Over the frequency range of 80 to 6300 Hz, the average sound absorption coefficients of the alkalized, nonwoven windmill-palm fabric, acetylated nonwoven fabric, and palm sheath samples were 0.31, 0.36, and 0.23, respectively.

To further verify the effect of the surface modification on the sound absorption properties of the windmill palm fibers, detailed statistical analysis was also performed; the results are shown in Table 4. The results show that prior to, and following the surface modification, there a significant difference in the sound absorption coefficient (P ≤ 0.05). Thus, we conclude that the alkali treatment, as well as the acetylation treatment, has a significant positive affect (Significance = 0.002) on the acoustic properties of the fibers. The acetylated fiber possesses nanoscale (10 to 50 nm) pores on its surface. Owing to this unique microstructure, the acetylated windmill palm fiber is considered a novel raw material for the preparation of sound-absorbing nonwoven fabrics. The nanoscale pore structure of the materials can significantly increase their specific area and surface permeability. This indicates that sufficient space exists for the transfer of sound. This consequently has a positive effect on acoustic insulation^28,29^. The mass of the acetylated sample is half that of the nonwoven windmill palm fabric sample, and its average sound absorption coefficient is 0.36. This information can be used for the development of novel, acoustic bio-cellulose materials.

**Table 4:** Results of one-way ANOVA test that examined the effects of the surface modification of the windmill-palm fibers on their sound absorption properties.

Project	Sum of squares III	d_f_	Mean square	F	Significance
Correction model	4.561	21	0.217	28.894	0.000
Intercept	6.936	1	6.936	922.696	0.000
Frequency	4.451	19	0.234	31.167	0.000
Sample	0.110	2	0.055	7.300	0.002
Error	0.286	38	0.008		
Sum	11.783	60			
Correction of a total	4.847	59			

## Discussion

The chemical modification results in the substitution of the OH groups of the cell walls of the cellulosic fibers with CH_3_CO groups^12^. The reduction in the number of OH groups results in the destruction of the connections among the cellulose components. This results in the development of a rough surface with pores of various sizes. The reduction in the number of OH groups also results in lower moisture absorption, a lower degree of swelling, and reduces the presence of voids at the interface^30^. It also has a positive effect on the stress transfer from the matrix to the fiber^31,32^.

Owing to the acetylation treatment, the surface of the single windmill palm fiber became hydrophobic. Under high-humidity environmental conditions, the hydrophobic surfaces are not wetted by the liquid. Instead, air inclusions or vacuums are formed, which lead to lower energy losses compared with those of hydrophilic surfaces^33^. Owing to the pore structure formed by the random arrangement of fibers, the 47% hollow structure of each single fiber, and the presence of nanoscale pores on the cell walls of the fiber, the porosity level of the material changed from the nanometer to the micrometer scale. In addition, the nanoscale holes formed by the acetylation treatment increased the size of the space available for the transfer of sound and increased the roughness of the fiber-cell wall^29^. Owing to the viscous damping and thermal loss caused by their complicated pore structures, the nonwoven fibrous fabrics demonstrate superior sound absorption capability^34,35^.

## Materials and Methods

### Materials

The windmill palm sheath meshes were obtained from Mount Huang, Anhui province, China. The windmill palm fibers were treated with a 4 wt.% hydrogen peroxide (H_2_O_2_) and 1.5 wt.% sodium hydroxide (NaOH) solution, with a 1:50 fiber-to-extractant ratio (g/mL), at 85 °C for 4 h^5^. The fiber suspension for was stirred for about 2 min. Subsequently, separate, alkalized single windmill palm fibers were obtained. These single fibers were filtered and dried in an oven at 60 °C for 24 h.

Subsequently, 1 g of the dried, alkalized, single windmill-palm fibers was immersed in 20 mL of dimethyl formamide (DMF). This mixture was stirred occasionally for 20 min; subsequently, various amounts of acetyl chloride and various concentrations of pyridine were added. The resultant mixture was held for various periods and temperatures based on the results of the orthogonal design. The parameters of each test are shown in Table 5.

**Table 5:** The parameters of the acetylation treatment.

Samples	Acetylchloride/vt.%	Pyridine/vt.%	Temperature/°C	Time/h
1	7.5	2.5	30	4
2	7.5	5.0	40	6
3	7.5	7.5	50	8
4	12.5	2.5	40	8
5	12.5	5.0	50	4
6	12.5	7.5	30	6
7	17.5	2.5	50	6
8	17.5	5.0	30	8
9	17.5	7.5	40	4

**Figure 6:**
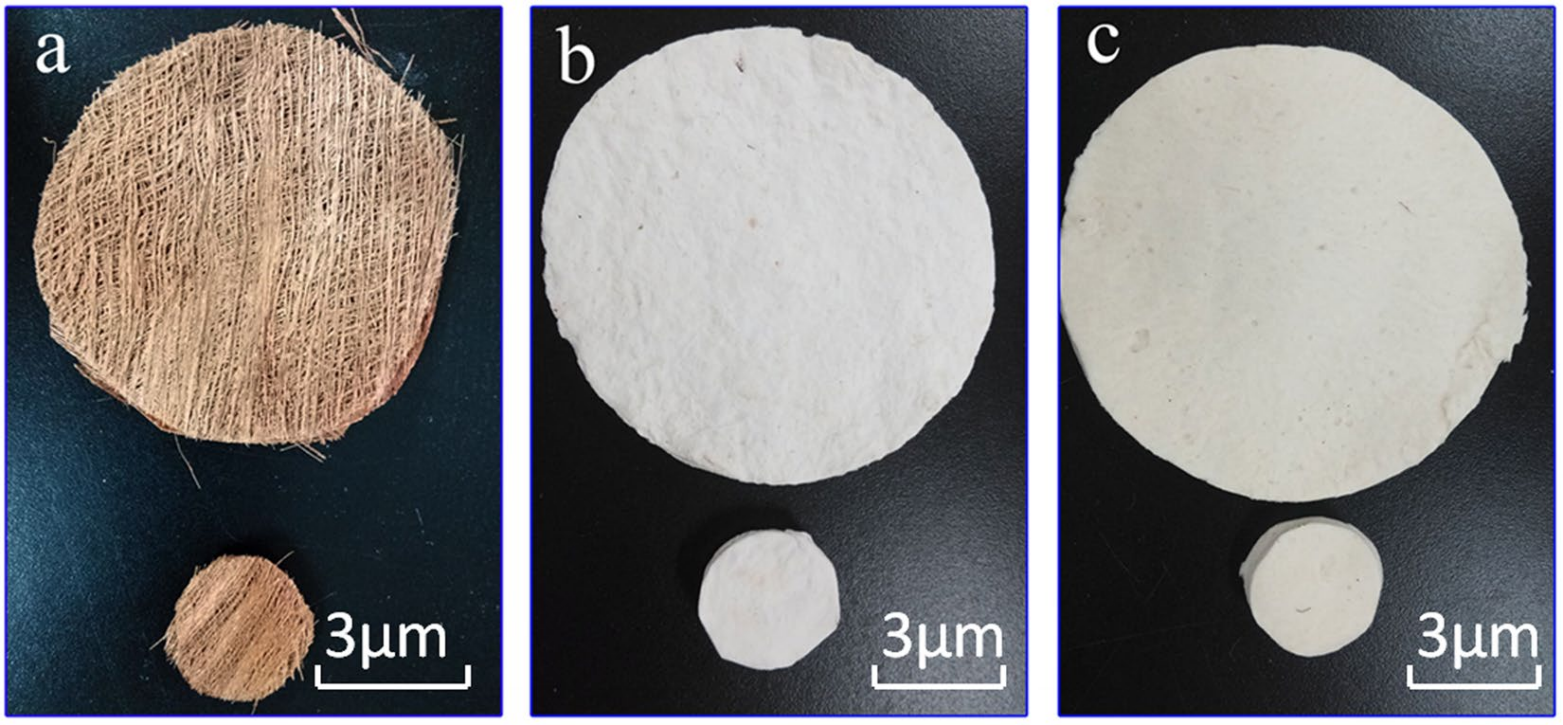
Samples of (**a**) windmill palm mesh, (**b**) alkalized, nonwoven single fiber fabric, and (**c**) acetylated nonwoven fabric.

Following the acetylation treatment, the fibers were washed with distilled water, dried at 60 °C, and stored. The alkalized and acetylated single windmill-palm fibers were obtained. The nonwoven fabric, shown in Fig. 6, was prepared using a wet laying technique^29^.

### Contact-angle measurement

A contact-angle goniometer (DSA 100 S, KRUSS, Germany) was employed to measure the contact angle of the nonwoven windmill palm fabric. A dosing volume of 3 μL of water was placed onto the surface of each sample, and the contact angle was measured. This procedure was repeated three times.

### Characterization of single windmill-palm fibers

The surfaces of the alkalized and acetylated single windmill-palm fibers were observed using scanning electron microscopy (SEM; S-4800, HITACHI, Japan). The samples were gold spluttered for 20 s. An accelerating voltage of 5 kV was used, along with a working distance of 8.9 mm.

### Moisture regain test

The moisture regains values of the windmill palm fibers and single fibers were determined using the method described in standard GB/T 9995-1977. About 1.0 g of the fiber samples was dried at 105 °C in a hot-air oven for 4 h. The weight of the dried fiber was denoted as *Wa*. The fibers were then allowed to regain moisture under a standard atmosphere of 65% RH and 20 °C. Initially, the weight of the fibers was recorded every 5 min, and subsequently, every 20 min until the fibers achieved moisture sorption equilibrium. The weight of each of the samples, at a specific time, was recorded as *Wi*. The moisture regain value was determined by the following equation (Eq. (1)):

1 $$Moisture\,regain({\rm{ \% }})=(Wa-Wi)/Wa\ast 100$$

### Sound absorption measurement

The normal incidence absorption coefficient was determined using two microphone impedance tubes (SW463, Shengwang, China). This was achieved by employing the transfer function method over the frequency range of 80 Hz to 6300 kHz. The average sound absorption coefficient was calculated by determining the mean of the absorption coefficients at 125, 250, 500, 1000, 2000, and 4000 Hz.

## Conclusions

In summary, hydrophobic windmill-palm fibers, with a moisture regain value of 3.86% and contact angle of about 143 ± 4°, were prepared by performing an acetylation treatment at 50 °C for 8 h, with 12.5 vt.% acetyl chloride and 7.5 vt.% pyridine. The acetylated fiber possessed nanoscale (from 10 to 50 nm) pores on its surface. Owing to its unique microstructure, the acetylated windmill-palm fiber can be considered a novel raw material for the preparation of sound-absorbing nonwoven fabrics. The weight of the acetylated fabric is half that of the nonwoven windmill palm fabric, and its average sound absorption coefficient is 0.36, which is even higher than that of the nonwoven windmill palm fabric (0.31). The results of this study can be used for the development of novel, acoustic bio-cellulose materials.
